# Training peer researchers for CBPR on racism in health care in Germany: Tanja Gangarova

**DOI:** 10.1093/eurpub/ckac129.610

**Published:** 2022-10-25

**Authors:** T Gangarova

**Affiliations:** National Discrimination and Racism Monitor, DeZIM-Institut, Berlin, Germany

## Abstract

For many affected by ongoing coloniality, research is not neutral or objective, but is part of the colonial violence inflicted through disciplines as diverse as medicine and ethnography. Against this background, choosing Community-based Participatory Research (CBPR) as research strategy for researching racism in health care can be seen as a way of challenging existing epistemologies of ignorance by embracing diversity of knowledge production. ‘Community Perspectives on Racism in Health Care’ is a participatory research project carried out in the frame of the National Discrimination and Racism Monitor which aims to improve the involvement of people affected by racial injustice in the research process, collect data on and generate theory about healthcare-related racism. It is conducted by DeZIM-Institut in collaboration with different racialized communities. Community members were trained as peer researchers and supported to conduct CBPR-projects. Two CBPR-projects were conducted: 1) digital focus groups with people marked as Muslims 2) digital focus groups with Black people. ‘Racial’ health disparities were identified on three levels: differential access to health care, differential care within the system and differences in exposures. The theory was built inductively, while drawing on existing theoretical concepts from the disciplines of public health, postcolonial and decolonial studies. Racialized people used the opportunity to be trained as peer researchers. They conducted CBPR projects tailored to the needs of their communities and conceptualized racism in the broader context of health care. Suggestions were made for improving health care services. Including those most impacted by racial injustice as peer researchers enables us to widen the “we” who constitute the researchers and to harvest the wisdom of situated knowledges. However ongoing challenges have been faced raising the question of the (im)possibilities of democratic knowledge production in German academia.

**Key messages:**

• Including those most impacted by racial injustice as peer researchers enables us to harvest the wisdom of situated knowledges.

• Ongoing challenges have been faced by trying to establish democratic knowledge production in German academia.

